# FTD-PSP is an Unusual Clinical Phenotype in A Frontotemporal Dementia Patient with A Novel Progranulin Mutation

**DOI:** 10.14336/AD.2021.0309

**Published:** 2021-10-01

**Authors:** Bin Deng, Zhe Zheng, Jialing Zheng, Wanlin Yang, Yu Huang, Yuqi Luo, Dana Jin, Lu Shen, Kunlin Jin, Qing Wang

**Affiliations:** ^1^Department of Neurology, Zhujiang Hospital of Southern Medical University, Guangzhou, Guangdong 510282, China.; ^2^Department of Neurology, Xiangya Hospital, Central South University, Changsha, China.; ^3^College of Biological Sciences, University of California, Davis, CA 95616, USA.; ^4^Department of Pharmacology and Neuroscience, University of North Texas Health Science Center, Fort Worth, TX 76107, USA

**Keywords:** Progranulin, Parkinson Syndrome, progressive supranuclear palsy, frontotemporal dementia, mutation

## Abstract

Progranulin (GRN) mutations are a major cause of frontotemporal dementia (FTD); the spectrum of clinical phenotypes of FTD is much more extensive than previously reported. The frequency and locations of GRN mutations in Chinese patients with FTD remain uncertain. We performed cDNA sequencing in one sporadic male patient who initially presented FTD symptoms. Brain magnetic resonance imaging (MRI) and positron emission computed tomography/computed tomography (PET/CT) were applied to further confirm the diagnosis of FTD from this patient. Cellular apoptosis and survival test were performed to identify the function of GRN. We identified one novel missense GRN mutation (c.1498G>A, p.V500I) in this patient, who initially presented typical behavioral-variant frontotemporal dementia (bvFTD) features but then presented progressive supranuclear palsy (PSP) clinical characteristics 5 years after onset. Besides, WT GRN protein showed an adequate trophic stimulus to preserve the survival of SH-SY5Y cells in the medium free of serum, while GRN mutation (c.1498G>A, p.V500I) may impair the ability of supporting cell survival. This study owns significant implications for genetic counseling and clinical heterogeneity. We illustrate the fact that FTD presenting features of bvFTD and PSP in one patient could be considered as a specific phenotype in patients with GRN mutations. GRN p.V500I led to the neuronal degeneration *in vitro*; this finding provides a significant evidence that this mutation may be a new causative mutation in patients with FTD.

The progranulin gene (GRN) encodes a 75-80 kDa secreted growth factor granulin precursor (progranulin) [1, 2]. GRN mutations, which are considered a major cause of familial frontotemporal lobar degeneration (FTD), explaining 25% of FTD cases worldwide [3-7], lead to the shortage of granulins, and GRN mutations are also found in other neurodegenerative diseases, such as Alzheimer’s disease (AD), corticobasal syndrome (CBS) and other atypical parkinsonian disorder (APD) patients [3, 8-13]. The spectrum of clinical presentations of FTD related to mutations in GRN is highly heterogeneous, including gestural apraxia, parkinsonism, abnormalities of behavior and personality, language impairments, and visual hallucinations, as present in 25-40% of patients [3, 8, 14-19]. Different clinical subtypes of FTD are described in Fig 1 and Table 1.

**Figure 1.**
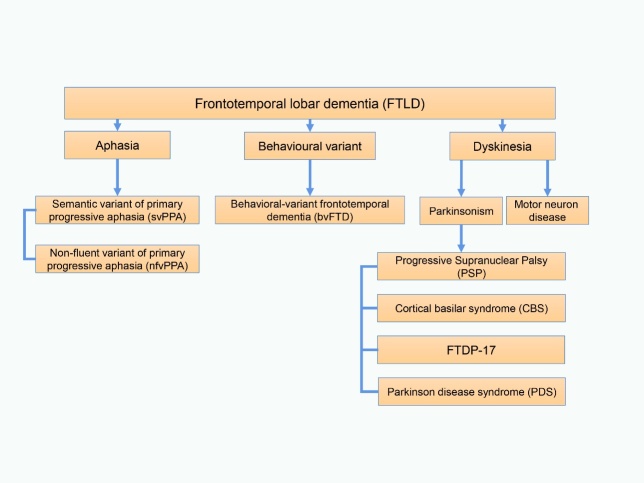
Different clinical subtypes of FTD.

Although up to 50% of patients with FTD report a family history of dementia, among which behavioral-variant frontotemporal dementia (bvFTD) has the highest prevalence among FTD clinical syndromes and takes up for nearly 70% of all FTD cases [20-24], some patients with FTD display sporadic forms. The clinical heterogeneity in familial and sporadic forms of FTD is remarkable, with patients illustrating different kinds of mixtures of disinhibition, dementia, progressive supranuclear palsy (PSP), corticobasilar syndrome (CBS), and motor neuron disease (MND). Of note, FTD may precede, follow or coincide with the onset of motor symptoms, demonstrating the clinical heterogeneity of FTD.

**Table 1:** Different clinical subtypes of patients with GRN mutations in published literatures.

	Aphasia		Behavioural variant	Dyskinesia		References

	nfvPPA	svPPA	bvFTD	Parkinsonism	MND	
Case1-20	+	NA	NA	NA	NA	[33-51]
Case 21-24	NA	+	NA	NA	NA	[33, 52-54]
Case 25-49	NA	NA	+	NA	NA	[36, 39, 41, 46, 53, 55-72]
Case 50-61	NA	NA	NA	+(CBS)	NA	[38, 47, 53, 60, 61, 73-79]
Case 62-65	NA	NA	NA	+(PSP)	NA	[66, 80-82]
Case 66-68	NA	NA	NA	+(PDS)	NA	[46, 51, 83]
Case 69	NA	NA	NA	+(DLB)	NA	[84]
Case 70	NA	NA	NA	+(FTDP-17)	NA	[85]
Case 71-74	NA	NA	NA	+	NA	[66, 86-88]
Case 75-77	NA	NA	NA	NA	+	[65, 66, 89]
Case 78-82	+	NA	+	NA	NA	[63, 73, 86, 90, 91]
Case 83-84	NA	NA	+	+	NA	[69, 92]
Case 85	NA	NA	+	+(PSP)	NA	[72]
Case 86-87	+	NA	NA	+	NA	[93, 94]
Case 88	+	NA	NA	+(CBS)	NA	[95]
Case 89	+	NA	+	+	NA	[93]

NA: not available; nfvPPA: Non-fluent variant of primary progressive aphasia; svPPA Semantic variant of primary progressive aphasia; bvFTD: Behavioral-variant frontotemporal dementia; MND: Motor neuron disease; PSP: Progressive Supranuclear Palsy; CBS: Cortical basilar syndrome; FTDP-17: FTD with parkinsonism linked to chromosome 17q; PDS: Parkinson’s disease syndrome; DLB: dementia with lewy bodies.

While GRN mutations (OMIM *138945, Arg493X, granulin precursor, Leu271LeufsX10) are responsible for some cases of familial FTD, most of these mutations were observed in Caucasian people [6, 8, 25]. The frequency and locations of GRN mutations in Asian populations are far less known [1, 26-28]. In this study, we intended to 1) recognize novel missense GRN mutations in one sporadic patient with FTD by cDNA sequencing; 2) define their clinical phenotypes and heterogeneity; and 3) summarize various FTD clinical phenotypes with different novel GRN mutations through literature review. Our results offer significant clues to geneticists on the pathogenesis of FTD in the Chinese population.

**Table 2:** Summary of clinical manifestations and auxiliary examinations of the patient with GRN mutations.

	patient
Age of onset	55
Symptoms at onset	Progressive memory deterioration
Duration(years)	8
GRN mutation	c.1498G>A; p.V500I (heterozygous)
Clinical diagnosis	FTD
Family history	No
Ocular motor dysfunction	Vertical supranuclear gaze palsy
Postural instability	No
Akinesia	Bradykinesia, rigidity and postural tremor of right upper limb
Cognitive dysfunction	Progressive memory deterioration
Dysarthria	No
Dysphagia	YES
Autonomic function	Polyuria and urinary incontinence occasionally
MRI	A mild degree of cerebral atrophy
EEG	Mildly abnormal
PET/CT	Decreased dopamine metabolism in bilateral corpus striatum and mildly decreased glucose metabolism in bilateral frontotemporal lobe
Macula OCT	Local deficiency of retinal photoreceptors and cystic liquid area
MoCA	14/30
MMSE	18/30
NMSS	45
UPDRS	80
H&Y	3
Biomarkers in CSF	
Aβ1-42	892.22pg/ml
Aβ1-40	3999.62pg/ml
t-Tau	118.41pg/ml
p-Tau	17.56pg/ml
Aβ1-42/Aβ1-40	0.22

Aβ: amyloid β protein; H&Y: the modified Hoehn and Yahr staging scale t-Tau: total Tau protein; p-Tau: phosphorylated Tau protein; FTD: frontotemporal dementia; CSF: cerebrospinal fluid; EEG: electroencephalogram; GRN: progranulin; MMSE: Mini-Mental State Examination; MND: Motor Neuron Disease; MoCA: Montreal Cognitive Assessment; MRI: magnetic resonance imaging; NMSS: Non-Motor Symptoms Scale for Parkinson’s Disease; OCT: optical coherence tomography; PET/CT: positron emission computed tomography/computed tomography; UPDRS: The unified Parkinson’ Disease Rating Scale.

## MATERIALS AND METHODS

### Patients and clinical examination

One male patient proven to harbor different GRN mutations and initially suffering from cognitive impairment was recruited from the Department of Neurology, Zhujiang Hospital of Southern Medical University. He received detailed clinical examinations that were performed by two neurologists (one was a movement disorder specialist); several clinical scales were used to examine disease status(Table 2), including standardized ratings of disease severity using the modified Hoehn and Yahr (H&Y) staging scale, the Non- Motor Symptoms Scale for Parkinson’s Disease (NMSS), the Mini Mental State Examination (MMSE), the Unified Parkinson Disease Rating Scale (UPDRS), and the Montreal Cognitive Assessment (MoCA). The details of his clinical manifestations and cranial encephalography (EEG) were collected (Table 2). This study was approved by the ethics committees of the Zhujiang Hospital of Southern Medical University (2020- KY-018-02). Informed consent was obtained from the participant according to the Research Ethics Boards.

### Magnetic resonance imaging (MRI) and positron emission computed tomography/computed tomography (PET/CT)

Retrospective routine cranial MRI data from this patient were collected and re-evaluated by an experienced neuroradiologist. The area of the midbrain and pons was measured on the mid-sagittal MRI to get the assessment of the midbrain and pons atrophy; an area smaller than 88 mm2 or 417 mm2 was regarded as midbrain or pons atrophy, respectively. 18F-Fluorodeoxyglucose (18F-FDG) and 18F- fluorodihydroxy phenylalanine (18F-DOPA) PET/CT scans were performed on this patient in 2017. To assess glucose metabolism and dopamine metabolism in the brain, we estimated the uptake and distribution of 18F- FDG and 18F-DOPA. Scores for a PD-related brain pattern (PDRP) were also measured in 18F-FDG PET/CT scans.

### Gene sequencing of related diseases and cerebrospinal fluid samples

Whole-blood samples were extracted from the patient for genetic sequencing analyses. Known mutations in GRN, microtubule-associated protein tau (MAPT), chromosome 9 open reading frame 72 (C9orf72), valosin-containing protein (VCP), TAR DNA-binding protein (TARDP), charged multivesicular body protein 2B (CHMP2B), amyloid precursor protein (APP), presenilin 1 (PSEN1) and presenilin 2 (PSEN2) were all screened by polymerase chain reaction (PCR) and subsequent sequencing. Consent for gene sequencing of related diseases (PSP, FTD, AD and PD) was acquired from the patient and his family members. Gene sequencing of the patient was conducted by Mygenostics Inc. (Beijing, China) and Amcarelab Inc. (Guangzhou, China). Lumber puncture was performed to withdraw cerebrospinal fluid (CSF) samples for the measurement of β-amyloid and tau proteins using enzyme-linked immunosorbent assay (ELISA).

### Cell transfection

The Gateway recombination cloning technique was adopted to construct recombinant vectors highly expressing progranulin protein. GRN cDNA constructs, pRP[Exp]-EGFP/Neo-CMV>V5/6xHis/{Stuffer300}, pRP[Exp]-EGFP/Neo-CMV>hGRN[NM_002087.3]/V5/6xHis and pRP[Exp]-Neo-CMV>{hGRN[NM_ 002087.3]*(V500I)}/V5/6xHis were designed and ordered from the Vector Builder service (Guangzhou, China). SH-SY5Y cells that express human GRN were established by transfection transiently using LipofectamineTM 2000 transfection reagent (Lip2k, Invitrogen). Briefly, for 6-well plates, 4 μg of DNA and 10 μl of Lip2k (1:2.5 ratio) were firstly diluted into 250 μl Opti-MEM respectively, and incubated at room temperature for 5 min. Then, the diluted DNA and Lip2k were combined and incubated at room temperature for 20 min, the DNA-lipid complexes were added to the cells and incubated at 37°C [29]. To detect if mutant GRN affects the ability of supporting cell survival, 48 h later, the medium was changed to the medium free of serum and incubated at 37°C for another 72 h. After that, TUNEL assay, immunofluorescence and Cell Viability Test were performed to detect apoptosis and survival.

### Western Blot

SH-SY5Y cells were lysed with RIPA buffer contained Protease Inhibitor Mixture (Beyotime, Shanghai, China) and the proteins in the medium were also collected. The lysates were split completely with ultrasound incubating on ice to avoid unnecessary degradation. After adding 2-hydroxy-1-ethanethiol (diluted 1:20), the lysates were boiled for 10 min. Then the proteins were separated by gel electrophoresis. After being blocked for 45min at room temperature, the blots were incubated in TBST with 1:200 anti-human GRN antibody (mouse anti-human GRN) over-night at 4 ?. After extensive washing with TBST for three times, the blots were incubated with the secondary antibody (diluted 1:1,000) at room temperature for 45min. After being washed three times, the blots were visualized using enhanced chemiluminescence. It was the same way to deal with the proteins using mouse monoclonal β-actin antibody (diluted 1:1,000) as a control for total protein loading.

### Apoptosis TUNEL assay

Cells were fixed with 4% PFA for 20min. Then cells were incubated in permeabilization buffer (containing 0.2% Triton X-100 in PBST) for 20 min after being washed for three times in PBST to remove the extra PFA. After that, cells were rinsed extensively for three times with PBS. With the direction of the manufacturer’s instructions, the cells were stained with reaction solution from One Step TUNEL Apoptosis Assay Kit (Beyotime, Shanghai, China). After incubation at 37°C for 45min, cells were rinsed for three times using PBST and dyed with 4’,6-diamino-2-henylindole (DAPI) for 10 min avoiding light. After washing with PBST for three times, cells were mounted with sealing solution and observed with a Nikon microscope and apoptotic cells (TxRed) versus total cells (DAPI) were recorded by visible observation.

### Cell Viability Test

Briefly, media was carefully removed from the 96-well plate and 100μl serum-free medium with 10% CCK-8 reagent was added. After reacting for 90min at 37? avoiding light, the absorbance at 450nm of the cells was measured with a microplate reader.

### RESULTS

#### Clinical presentation and GRN mutation

**Figure 2.**
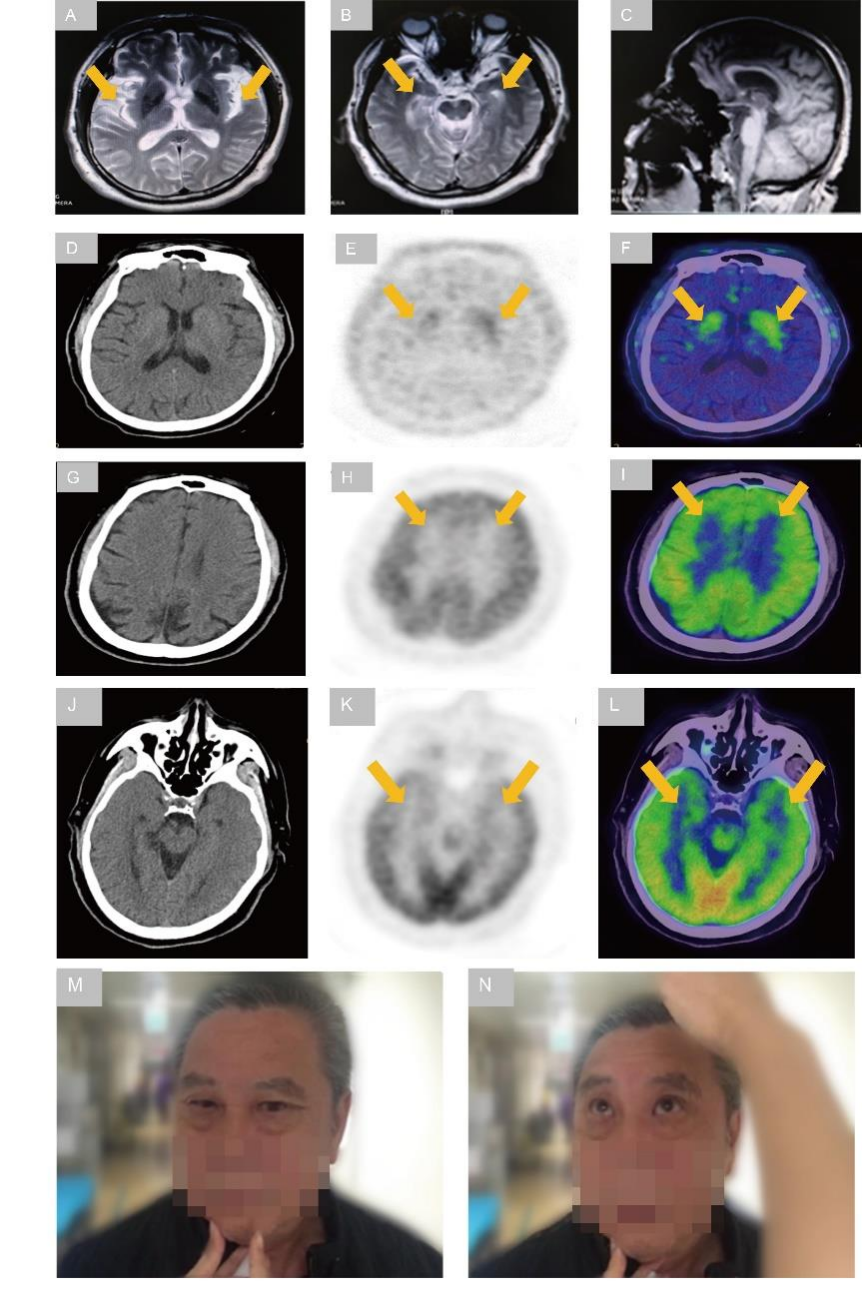
Neuroimaging characteristic of the patient with GRN mutations. (A and B) Axial T2-weighted images showing bilateral lateral fissure deepened and widened (arrow) and dilatation of temporal horn of lateral ventricle (arrow). (C) Sagittal T2-weighted images showing mild generalized cortical atrophy. (D-F) ^18^F-DOPA PET/CT scans showing profoundly reduced DOPA metabolism in bilateral corpus striatum (arrow). (G-I)^18^F-FDG PET/CT scans showing a symmetrically reduced glucose metabolism in bilateral frontal lobe (arrow). (J-L) ^18^F-FDG PET/CT scans showing a symmetrically reduced glucose metabolism in bilateral temporal lobe (arrow). (M) This photograph showing the impossibility of the patient to look downward with the guidance of a neurologist. (N) This photograph showing the difficulty of the patient to look upward with the guidance of a neurologist.

**Figure 3.**
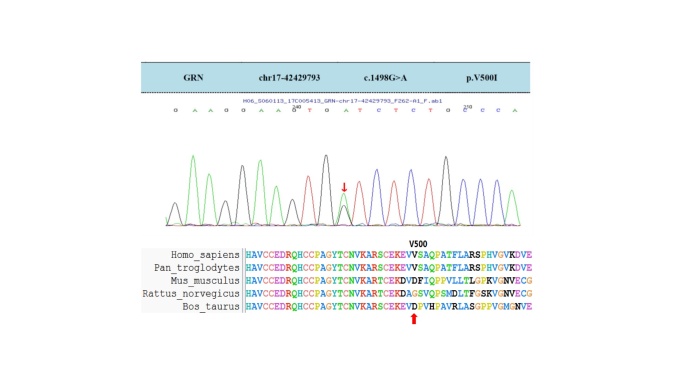
Genetic sequencing analyses.

The patient was a 62-year-old Chinese man with a four-year history of hypertension. Memory deterioration started in 2013 as short-term memory loss. He frequently forgot what he planned to do in a very short time frame. Neither parkinsonism nor other movement disorder symptoms were observed until 2016, when his memory worsened. He could not find his way home and forgot some important dates in his life, such as the birthday of his daughter and the date when his house was bought. He displayed parkinsonism symptoms, including bradykinesia, rigidity and postural tremor of the right upper limb since 2016, while he occasionally complained of polyuria and urinary incontinence. In 2018, he presented PSP-like clinical phenotypes such as vertical supranuclear gaze palsy and choking. With the treatment of levodopa/benserazide, pramipexole and memantine, memory deterioration and parkinsonism symptoms were controlled without obvious progression, but those symptoms were still recurrent and fluctuating. His family history was negative for dementia. Cranial MRI demonstrated a mild degree of cerebral atrophy (Fig 2.), and positron emission computed tomography/computed tomography (PET/CT) revealed bilateral decreased dopamine metabolism in the corpus striatum and bilateral mildly decreased glucose metabolism in the frontotemporal lobe. The score for the PD-related brain pattern (PDRP) measured in the ^18^F-FDG PET/CT scans of the patient was 16.8. In 2017, genetic testing was performed to recognize a novel missense GRN mutation (c.1498G>A; p.V500I; Fig 3.). Based on the clinical characteristics, MRI, PET and genetic mutation, this patient was clinically diagnosed with FTD. In 2019, macular optical coherence tomography (OCT) was performed to show the local deficiency of retinal photoreceptors in the lower-inner quadrant of the oculus sinister and cystic liquid area. Electroencephalography (EEG) showed mild abnormalities, with a slowdown of the basic rhythm. His MMSE score was 18/30, and his MoCA score was 14/30. The MoCA or MMSE scores indicated that the patient’s execution, attention, memory and visuospatial functions were impaired. In addition, his UPDRS score was 45, and his NMSS score was 80.

#### Mutant GRN-V500I loss the ability to support SH-SY5Y cell survival

**Figure 4.**
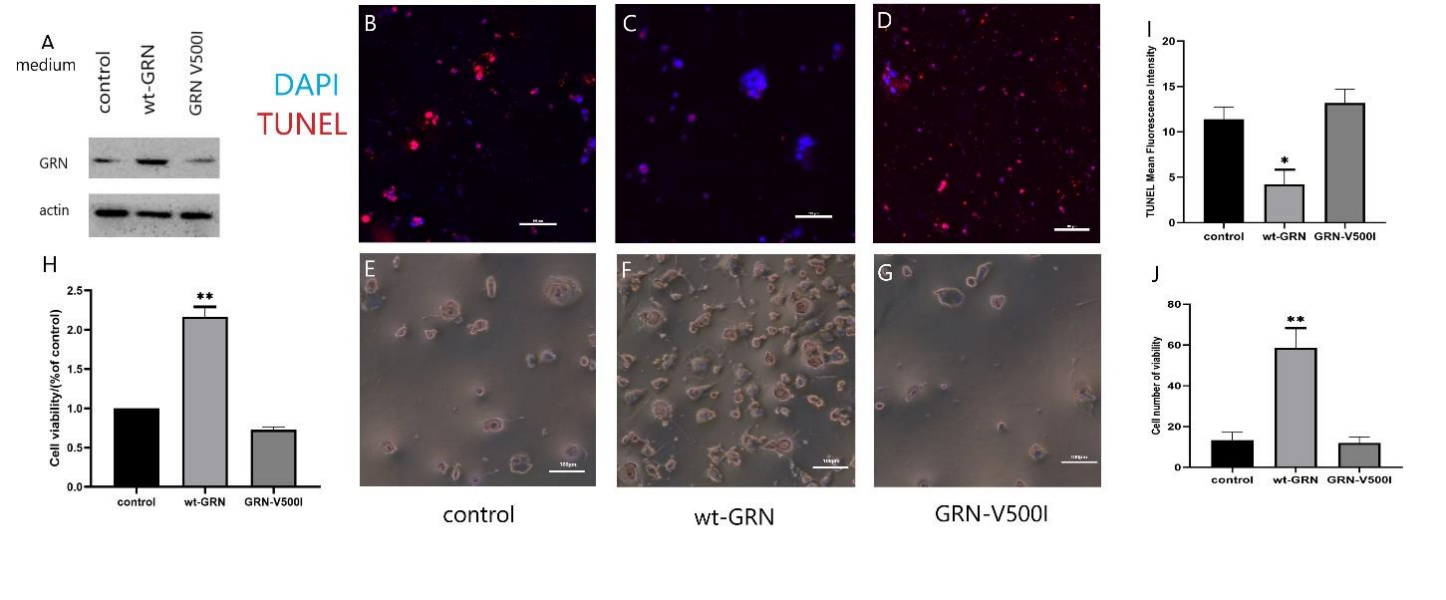
GRN mutation over-expression increases apoptosis of SH-SY5Y cells. Mutant GRN loss the ability to support SH-SY5Y cell survival after the removal of serum. (A) Western Blot analysis confirmed presence of GRN after transfection. (B, E) SH-SY5Y cells transfected with control vector only. (C, F) Transfected with WT hGRN. (D, G) Transfected with mutant V500I. (B-D) Micrographs showing the distribution of DAPI and TxRed. (E-G) Micrographs showing the survival of cells. (H) CCK-8 test showing the cell viability. (I) The quantitative result of fluorescence showing the cell apoptosis. (J) The quantitative result of cell number showing the cell viability.

To detect if mutant GRN affects the ability of supporting cell survival, SH-SY5Y cells were transiently transfected with control, WT and mutant V500I plasmids. 48 h later, the medium was changed to the medium free of serum and incubated at 37°C for another 72 h, TUNEL assay and immunofluorescence was performed to detect apoptosis. Through Western blot, the over-expression of the GRN protein was verified (Fig 4). 72h after the removal of serum, cell number showed a significant reduction in control and V500I cultures, while cultures of WT cells showed no such change. In cellular immunofluorescence, the apoptosis cells dyed with red fluorescence showed a significant increase in the control and V500I cultures versus the WT cultures. In addition, no obvious difference was observed in apoptosis cells between the control and V500I cultures (p value < 0.05, n=3; Fig 4). In cell viability test, there was a significant decrease in cell viability in the control and V500I cultures versus the WT cultures. In addition, no obvious difference was found in cell viability between the control and V500I cultures (p value < 0.05, n=3; Fig 4). These results suggest WT GRN protein is an adequate trophic stimulus to preserve the survival of SH- SY5Y cells in serum-free medium, while V500I may affect the ability of supporting cell survival.

### DISCUSSION

FTD, usually caused by GRN mutation, is a genetically complex neurodegenerative disorder with divergent and unexpected phenotypes and sometimes overlaps with CBS, PSP, multiple system atrophy (MSA), amyotrophic lateral sclerosis (ALS) or MND [3, 6]. T Heterozygous GRN carriers’ age at onset differs from 40 to 85 years. Here, we report one novel sporadic case with GRN- related disorders and GRN heterozygous missense pathogenic variants. Our findings indicated that different pathogenic mutations in GRN, in terms of missense and splice sites, may play important roles in FTD patients. The course in our patient presented two phases, i.e., behavioral-variant frontotemporal dementia followed by progressive supranuclear palsy symptoms. To our knowledge, few studies have shown various clinical features in one FTD patient.

First, by DNA sequencing, we identified one novel missense and heterozygous GRN mutation (c.1498G>A, p.V500I) in one sporadic patient with FTD; the genetic locus of GRN mutation in our case seems different from those in FTD cases in Western countries. In western countries, heterozygous mutations in GRN are a common cause of FTD [1, 6, 8], while GRN mutations are sparsely seen in patients in Eastern countries diagnosed with FTD [1, 30]. The majority of the pathogenic mutations are nonsense and splice-site mutations, which may lead to the loss of one GRN allele. However, similar to our case, missense mutations may also lead to the dysfunction of the GRN allele [3, 31]. Finch et al. reported two FTD patients with the nonsense GRN mutation c.1477C>T; p.R493X [3]. This mutation leads to a nucleotide change at nucleotide 1477, which is adjacent to the mutation site of our patient (c.1498G>A; p.V500I). However, the GRN mutation c.1477C>T; p.R493X is a nonsense mutation, resulting in the creation of a translation termination codon at amino acid 493 that brings about the partial damage of functional progranulin. The GRN mutation in our patient (c.1498G>A;p.V500I) is a missense mutation that cannot turn the corresponding amino acid into a stop codon, which is consistent with previous findings [1, 3, 32]. Chang et al., reported a missense mutation (p.T487I) of GRN in a female Asian patient with undefined APD and verified that the missense mutation (p.T487I) *in vitro* could be a causative mutation in patients with APD [1]. Similarly, Huin et al. reported a female patient diagnosed with neuronal ceroid lipofuscinosis type 11 (CLN11), which resulted from homozygous GRN mutations (c.1A>T; p.[Met1?]) and resulted in the entire loss of capability of the protein and presented a broad phenotypic spectrum [6]. Besides, our results (Fig 4) also suggest that WT GRN protein is an adequate trophic stimulus to preserve the survival of SH-SY5Y cells, while V500I may affect the ability of supporting cell survival. This *in vitro* finding further implies that the GRN mutant cause the neuronal degeneration.

Our literature search identified a spectrum of various clinical phenotypes in patients with FTD (Table 1), including the semantic variant of primary progressive aphasia (svPPA), Non-fluent variant of primary progressive aphasia (nfvPPA), behavioral-variant frontotemporal dementia (bvFTD), Parkinsonism and motor neuron disease (MND). Nevertheless, few studies have identified one patient with two different clinical phenotypes. More interestingly, our patient displayed clinical symptoms that were different from those in previous reports [1, 6, 32]. The initial clinical symptoms in our patient were typical FTD clinical features of the bvFTD subtype; 5 years later, he gradually presented PSP-like clinical phenotypes, including dysarthria, dysphagia, choking and vertical supranuclear gaze palsy, along with bilateral decreased dopamine metabolism in the corpus striatum and bilateral mildly decreased glucose metabolism in the frontotemporal lobe as demonstrated by PET-CT examination. Normally, MRI in GRN mutation carriers shows asymmetric frontotemporoparietal atrophy, as in our patient, while MRI in patients with PSP usually indicates atrophy in the mesencephalon. Collectively, when we encounter patients with GRN mutations who present atypical Parkinsonism syndrome such as PSP-like characteristics, we maintain that PSP-like syndrome should be differentiated from various stages of FTD and should be recognized as a specific phenotype of such genetic mutations.

In summary, this study has several implications and offers a better understanding of the genetic and molecular mechanisms of the GRN-related disorders as well as some significant clues for clinical practice. FTD presented bvFTD, and PSP-clinical phenotypic spectra in one individual patient would be considered as a specific clinical phenotype of GRN mutations in patients, i.e., typical FTD features followed by PSP symptoms in one individual case. We recognized a novel GRN mutation (c.1498G>A, p.V500I) from this patient. Our findings may be limited by the fact that this was a study that included only one patient. It may be significant to explore the molecular details of neurodegeneration in GRN mutations of a disease model founded by induced pluripotent stem cell technology.
